# Pangenome and immuno*-*proteomics analysis of *Acinetobacter baumannii* strains revealed the core peptide vaccine targets

**DOI:** 10.1186/s12864-016-2951-4

**Published:** 2016-09-15

**Authors:** Afreenish Hassan, Anam Naz, Ayesha Obaid, Rehan Zafar Paracha, Kanwal Naz, Faryal Mehwish Awan, Syed Aun Muhmmad, Hussnain Ahmed Janjua, Jamil Ahmad, Amjad Ali

**Affiliations:** 1 Atta-ur-Rahman School of Applied Biosciences (ASAB), National University of Sciences and Technology (NUST), H-12, Islamabad, Pakistan; 2 Research Center for Modeling and Simulation (RCMS), National University of Sciences and Technology (NUST), H-12, Islamabad, Pakistan; 3 Department of Computer Science and Information Technology, Stratford University, Falls Church, VA 22043 USA; 4 Institute of Molecular Biology and Biotechnology, Bahauddin Zakariya University, Multan, Pakistan

**Keywords:** *A. baumannii*, Pan-genome, Core genome, Epitopes, Vaccines

## Abstract

**Background:**

*Acinetobacter baumannii* has emerged as a significant nosocomial pathogen during the last few years, exhibiting resistance to almost all major classes of antibiotics. Alternative treatment options such as vaccines tend to be most promising and cost effective approaches against this resistant pathogen. In the current study, we have explored the pan-genome of *A. baumannii* followed by immune-proteomics and reverse vaccinology approaches to identify potential core vaccine targets.

**Results:**

The pan-genome of all available *A. baumannii* strains (30 complete genomes) is estimated to contain 7,606 gene families and the core genome consists of 2,445 gene families (~32 % of the pan-genome). Phylogenetic tree, comparative genomic and proteomic analysis revealed both intra- and inter genomic similarities and evolutionary relationships. Among the conserved core genome, thirteen proteins, including P pilus assembly protein, pili assembly chaperone, AdeK, PonA, OmpA, general secretion pathway protein D, FhuE receptor, Type VI secretion system OmpA/MotB, TonB dependent siderophore receptor, general secretion pathway protein D, outer membrane protein, peptidoglycan associated lipoprotein and peptidyl-prolyl cis-trans isomerase are identified as highly antigenic. Epitope mapping of the target proteins revealed the presence of antigenic surface exposed 9-mer T-cell epitopes. Protein-protein interaction and functional annotation have shown their involvement in significant biological and molecular processes. The pipeline is validated by predicting already known immunogenic targets against Gram negative pathogen *Helicobacter pylori* as a positive control.

**Conclusion:**

The study, based upon combinatorial approach of pan-genomics, core genomics, proteomics and reverse vaccinology led us to find out potential vaccine candidates against *A. baumannii.* The comprehensive analysis of all the completely sequenced genomes revealed thirteen putative antigens which could elicit substantial immune response. The integration of computational vaccinology strategies would facilitate in tackling the rapid dissemination of resistant *A.baumannii* strains. The scarcity of effective antibiotics and the global expansion of sequencing data making this approach desirable in the development of effective vaccines against *A. baumannii* and other bacterial pathogens.

**Electronic supplementary material:**

The online version of this article (doi:10.1186/s12864-016-2951-4) contains supplementary material, which is available to authorized users.

## Background

*Acinetobacter baumannii* is a Gram-negative coccobacillus responsible for nosocomial outbreaks and healthcare associated infections such as septicemia and pneumonia in the immuno-compromised patients [1]. During the last three decades, *A. baumannii* has emerged as one of the most difficult super-bug to treat in hospitals, worldwide [2]. It accounts for causing infections in about 12,000 patients annually and approximately 500 deaths in the United States, only [3]. The alarming characteristic of this pathogen is its ability to develop diverse mechanisms of resistance to existing drugs ultimately leading to therapeutic failures [4]. Recently, Infectious Diseases Society of America (IDSA) has included *A. baumannii* in the hit-list of top six priority pathogens, thus requiring a prompt response by the healthcare community to curb this pathogen [5, 6]. The massive challenge posed by multi and pan-drug resistant *A. baumannii* in the current post antibiotic era requires aggressive exploration of other solutions [7].

Besides active infection control measures and antibiotic stewardship programs, vaccination approach is predominantly reckoned as an alternative therapy to reduce the burden of infections caused by this pathogen [8]. Nevertheless, sequence- based reverse vaccinology approaches presented a more rational methodology to vaccine design since the discovery of universal vaccine against serogroup B meningococcal (menB) disease in 2000 [9, 10]. These *in silico* strategies led to comprehensive analysis of the pathogen genomics, proteomics, essential molecular pathways, virulence factors and host-pathogen interactions [11].

**Fig. 1:**
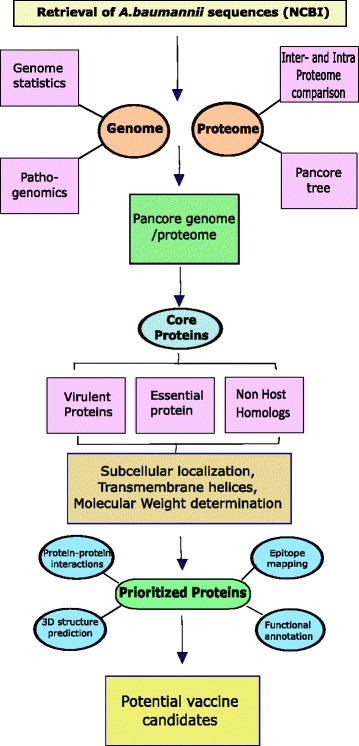
Schematic flow chart for the identification of putative core vaccine candidates in *Acinetobacter baumannii.* Sequences retrieved from NCBI were explored to get insight into the pan- and core genome of the strains. The evolutionary relationship between strains based on 16srRNA sequences was estimated. An elaborative inter- and intra-proteome comparison was executed followed by pan-core tree generation. Core proteome was subjected to reverse vaccinology approaches (sub-cellular localization, molecular weight estimation, essentiality, virulence, non-host homologs and trans-membrane helices) in order to prioritize efficient vaccine targets against *A. baumannii*. Subsequently, functional annotation of potential targets, the surface topology of predicted epitopes and their interactions with other proteins was determined

The global and rapid emergence of resistant *A. baumannii* necessitates a comprehensive and integrated study to find an alternative solution to this immediate threat. Since *A. baumannii* strains share a relatively high conserved features (genomic content) [12], therefore, pan-genome analysis could be a suitable strategy to analyze the available genomic data (genomes) of *A. baumannii* to explore the possible conservations and genomic diversity. In this study, we have analyzed all available complete genome sequences of *A. baumannii* strains and estimated the pan-genome with an aim to identify potential conserved immunogenic targets. To predict the promising broad spectrum vaccine candidates, we have explored the conserved genome which was subsequently analyzed for essential, virulent and antigenic proteins lying within the exo-proteome or secretome [13, 14]. Proteins substantiating to have a potential for vaccine candidates were then subjected to epitope mapping to predict the exposed antigenic epitopes possessing the ability to bind with both MHC I and II molecules. The schematic flowchart in Fig. 1 illustrates the strategy followed in this study incorporating combinatorial computational approaches, which could also provide an effective platform for the identification of vaccine targets against other pathogens.

## Methods

### Selection of genomes and genes predictions

To have a comprehensive overview of the species *A. baumannii*, the study included all the available completely sequenced genomes of *A. baumannii* strains*,* till the writing of the manuscript. The drafts and incomplete genomes were excluded to have standardization in the analysis. Genbank files were retrieved from NCBI Genbank (http://www.ncbi.nlm.nih.gov/genome) (NCBI Genome, RRID: SCR_002474). The DNA sequences of both the chromosome and plasmids in fasta format were retrieved and merged for each genome. For the prediction of genes and proteins the program Prodigal (Prokaryotic dynamic programming gene finding algorithm) was used. Prodigal was considered for its additional features such as increased sensitivity and specificity in gene prediction with improved translation initiation site recognition and reduced false positive predictions [15]. Table 1 illustrates the genomic data including accession numbers of chromosomes and plasmids, genome size, GC content and numbers of genes provided by NCBI and the one predicted by Prodigal.

**Table 1:** Genome statistics of completely sequenced *A. baumannii* strains

Pan- and Comparative Genomics of *Acinetobacter baumannii*										
#	Organism (Strain)	Chromosome/Plasmid	Size (Mb)	GC%	Genes/Proteins	Genes Predicted	New Genes	New Families	Pan-Genome	Core-Genome
1	*A. baumannii BJAB07104*	NC_021726.1/p1BJAB07104:NC_021727.1;p2BJAB07104:NC_021728.1	4. 0	38.95	3878/3754	3822	3822	3597	3597	3597
2	*A. baumannii MDR-TJ*	NC_017847.1/pABTJ1:NC_017848.1;pABTJ2:NC_020524.1	4.153	39.07	4014/3890	3956	286	268	3862	3454
3	*A. baumannii MDR-ZJ06*	NC_017171.1/pMDR-ZJ06:NC_017172.1	4.011	39.04	3847/3711	3799	129	123	3984	3272
4	*A. baumann;ii TYTH-1*	NC_018706.1	3.957	39.00	3754/3628	3697	76	76	4057	3176
5	*A. baumannii AC30*	CP007577.1/pAC30a:CP007578.1;pAC30b:CP007579.1; pAC30c:CP007580.1	3.922	38.82	3729/3660	3733	155	147	4203	3145
6	*A. baumannii BJAB0715*	NC_021733.1/pBJAB0715:NC_021734.1	4.053	38.91	3897/3756	3845	377	359	4560	2940
7	*A. baumannii BJAB0868*	NC_021729.1/p1BJAB0868:NC_021730.1; p2BJAB0868:NC_021731.1;p3BJAB0868:NC_021732.1	4.005	38.93	3820/3689	3762	55	55	4610	2899
8	*A. baumannii ZW85-1*	NC_023028.1/ZW85p2:NC_023031.1;pAbNDM-1:NC_019985.2	3.925	39.20	3684/3553	3625	237	234	4836	2847
9	*A. baumannii AC29*	NZ_CP007535.2	3.851	39.00	3659/3581	3613	11	10	4842	2834
10	*A. baumannii AB031*	NZ_CP009256.1	3.803	39.20	3595/3472	3521	241	224	5060	2754
11	*A. baumannii AB030*	NZ_CP009257.1	4.335	39.00	4336/4155	4285	549	334	5387	2715
12	*A. baumannii Abh12 OA2*	NZ_CP009534.1	3.875	39.10	3674/3563	3609	74	73	5454	2700
13	*A. baumannii LAC-4*	NZ_CP007712.1/pABLAC1:NZ_CP007713.1;pABLAC2:NZ_CP007714.1	3.968	38.98	3821/3683	3768	194	194	5640	2645
14	*A. baumannii 6411*	NZ_CP010368.1/p6411-66.409 kb:NZ_CP010903.1;p6411-89.111 kb:NZ_CP010369.1;p6411-9.012 kb:NZ_CP010370.2	4.061	38.75	3840/3686	3780	408	403	6038	2580
15	*A. baumannii 6200*	NZ_CP010397.1/p6200-114.848 kb:NZ_CP010398.1;p6200-47.274 kb:NZ_CP010399.1; p6200-9.327 kb:NZ_CP010400.1	4.073	39.18	3876/3762	3823	193	193	6230	2541
16	*A. baumannii NCGM 237*	NZ_AP013357.1	4.021	39.10	3894/3783	3838	93	93	6319	2527
17	*A. baumannii IOMTU433*	NZ_AP014649.1/pIOMTU433:NZ_AP014650.1	4.190	39.11	3999/3894	3926	242	237	6553	2527
18	*A. baumannii A1*	NZ_CP010781.1/pA1-1:NZ_CP010782.1	3.917	39.28	3735/3634	3650	140	134	6684	2513
19	*A. baumannii AB5075-UW*	NZ_CP008706.1/p1AB5075:NZ_CP008707.1/p2AB5075:NZ_CP008708.1; p3AB5075:NZ_CP008709.1	4.066	39.04	3929/3827	3867	89	70	6751	2511
20	*A. baumannii XH386*	NZ_CP010779.1/pAB386:NZ_CP010780.1	4.199	39.16	4062/3961	3991	27	27	6778	2511
21	*A. baumannii Ab04- MFF*	NZ_CP012006.1/pAB04-1:NZ_CP012007.1/pAB04-2:NZ_CP012008.1	4.192	38.91	4067/3944	4009	84	82	6856	2505
22	*A. baumannii ACICU*	NC_010611.1/pACICU1:NC_010605.1;pACICU2:NC_010606.1	3.996	38.90	3802/3613	3782	80	80	6927	2496
23	*A. baumannii AtCC MFF*	NZ_CP012004.1/pAB3:NZ_CP012005.1	4.006	38.94	3797/3688	3729	145	143	7066	2485
24	*A. baumannii AB307-0294*	NC_011595.1	3.760	39.00	3511/3363	3467	67	67	7127	2482
25	*A.baumannii AB0057*	NC_011586.1/pAB0057:NC_011585.1	4.059	39.18	3890/3669	3879	104	101	7218	2473
26	*A. baumannii AYE*	NC_010410.1/p1ABAYE:NC_010401.1; p2ABAYE:NC_010402.1; p3ABAYE:NC_010404.1; p4ABAYE:NC_010403.1	4.048	39.33	3878/3725	3795	54	48	7262	2473
27	*A. baumannii D1279779*	NC_020547.2/pD1279779:NC_020525.1	3.711	38.98	3523/3381	3473	88	87	7341	2459
28	*A. baumannii 1656-2*	NC_017162.1/ABKp1:NC_017163.1;ABKp2:NC_017164.1	4.023	39.08	3882/3733	3837	51	48	7385	2459
29	*A. baumannii TCDC-AB0715*	CP002522.2/p1ABTCDC0715;p2ABTCDC0715	4.218	38.89	4010/3956	4054	37	32	7408	2458
30	*A.baumannii ATCC 17978*	NC_009085.1/pAB1:NC_009083.1; pAB2:NC_009084.1	4.001	38.88	3469/3367	3951	212	210	7606	2445

NCBI database was accessed to retrieve the chromosomal and plasmid sequences of the included strains. Genome size, GC content, number of genes and proteins contained in an isolate were estimated. Genes/proteins in all the included strains were predicted by using Prodigal. Comparative genomics approaches were employed to estimate the pan- and core genome, new genes and gene families among the strains. The number of genes and proteins were compared, and also the size of the pan and core along with strain specific gene families are identified

### Genome organization and pathogenomics

To explore the genetic relatedness and evolutionary relationships, the selected strains were evaluated for their respective clonal lineage through previously published literature and phylogentic analysis [16, 17]. Clonal studies play a vital role to get insight about epidemiology of isolates and aid to control outbreaks both at the institutional level and global level [18]. Drug resistant strains of *A. baumannii* categorized as international clone (IC) I, II and III resulted in devastating outbreaks at global level [19, 20]. According to publicly available Pasteur’s MLST scheme (http://pubmlst.org/abaumannii/), IC II is considered as the largest clone in the global population, followed by I CI with distribution in more than 30 countries, worldwide [21]. The included strains were analyzed for the clonal distribution of virulence factors through Virulence factor database (VFDB) RRID:SCR_007969 [22]. Acquisition of virulence factors supports the bacteria to invade the host, evade the host defenses and persist in the host environment [23, 24]. With the aim to get insight into the virulence and pathogenic potential of *A. baumannii,* the virulence factors present in core genome were explored by employing VFDB and MvirDB [22, 25].

### Phylogenetic analysis

To better understand the evolutionary relationships and genomic variations, the *A. baumannii* genomes were analyzed through phylogenetic tree. The 16SrRNA genes were predicted by program RNAmmer [26]. Since 16SrRNA gene exhibit slower rate of evolution and are considered as hotspot for mutation, therefore frequently targeted for estimation of phylogenetic relationships between bacterial strains. Those sequences with reliable score of more than 1700 were considered for further evaluation [27]. ClustalW (RRID:SCR_002909) [28] was used for the multiple alignment of 16SrRNA sequences. MEGA6 [29] was used to create the phylogenetic tree based on Neighbor-Joining method [30]. 1000 bootstrap re-samplings were performed to estimate the consensus tree.

### Pan- and Core genome estimation

Pan-genome represents the entire gene repertoire and encompasses core genome and dispensable genome. Core genome comprised of genes that are present in all genomes of a given species, and are essential for bacterial growth, while dispensable sequences do not necessarily present in all genomes, and may be responsible for strain specific functions like pathogenicity, stress, resistance etc. [31] *A. baumannii* conserved core genome was estimated based on BLAST similarities between the genomes following previously established 50/50 rule [32–34]. Blast hit was considered significant when 50 % of alignment (amino acid) was identical where the length of alignment was 50 % of the longest gene in the comparison. According to this criterion, genes were clustered together in gene families if their amino acid sequences were at least 50 % identical. Multiple genes may also make a single gene family if they follow the same 50/50 rule. Similarly, all genes were grouped into gene families. The genes that did not fall in any gene family were assigned to their own unique gene family. Gene families having at least one gene in common among them were gathered into the core genome. Rest of the genes that did not fit into criteria may go to the species pan-genome [35–37].

### Intra- and inter-proteome comparisons

All of the selected genomes were translated into their proteomes to explore proteomic conservation among the strains. Pair-wise comparison of proteins was performed and visualized in the form of a matrix [38]. BLASTp comparison was carried out for all the proteins in one strain to all the proteins in other strains included in the dataset; and the association was estimated based on 50/50 rule as previously employed [32, 33]. Each corresponding box in the matrix represents the number of shared proteins and percent homology. For the comparison of any two genomes, protein families were built through single linkage clustering, so that each shared connection must be between sequences from different genomes (dark green color represents more homology between proteomes as shown in Fig. 4). In the matrix, an internal hit significantly similar to query protein was grouped into the same gene family. The bottom row in the matrix depicts the number of proteins that have homologous hits within the proteome itself (internal paralogs, shaded in red color, from 2.9 % least to 7.0 % highest illustrated in Fig. 4). Different levels of homology between the sequences were represented by different color intensities in the matrix (Fig. 4). Pan-core tree was generated based upon shared gene families between the strains. The relative Manhattan distance showed the evolutionary distance between the strains.

### Functional categorization of the core proteome

To prioritize potential core vaccine candidates, the core genome of all *A. baumannii* (30 genomes) strains was explored for the presence of essential genes using Database for Essential genes (DEG) [39]. Parameters set for BLAST against DEG were: E-value cut off 1e-10 and minimum bit score 100. Essential genes of bacteria are an effective therapeutic target, especially when bacteria confer multidrug resistance [40]. The core proteome was aligned with human proteome to pool out the human homologs, so as to eliminate the chances of autoimmunity [41]. Proteins having percentage identity <35 % and E-value <0.005 were considered as non-host bacterial proteins. Subsequently, virulent proteins were also estimated within core proteome by using Virulence factor database (VfDB) [22] and microbial virulence database (MvirDB). Blastp search was carried out against all the virulence associated protein by employing the following parameters: Bit Score > 100, E-Value < 1.0 e-5 and percentage Identity > 35 %. The database VFDB contains substantial information of virulence factors from 30 important bacterial pathogens (including *Acinetobacter species*), virulence associated genes, pathogenecity islands, protein structure and function characteristics. MvirDB is another comprehensive database for the identification of virulence factors, antibiotic resistance genes and protein toxins. It retrieves data from eight public access sequence databases and facilitates in rapid characterization of sequences related to virulence and pathogenesis [25]. PsortB was used to analyze the sub-cellular localization of proteins [42]. PsortB helps in sorting out the bacterial proteins as cytoplasmic, extracellular, periplasmic, outer and inner membrane. Proteins located in extracellular, periplasmic and outer membranes were preferred as effective vaccine candidates [43]. Topology Data Bank of Transmembrane Proteins, RRID:SCR_007964 (HMMTOP) was used to predict the localization of helical trans-membrane segments and topology of transmembrane proteins [44]. Proteins with ≤1 trans-membrane helices were selected as appropriate vaccine target as it is easy to purify, clone and express them [45]. Eventually, those proteins which were essential, virulent, non-host homologs, exposed or secreted with ≤1 trans-membrane helices prioritized as vaccine candidates. The molecular weight of the prioritized proteins was estimated by employing Expasy PI/MW tool (RRID:SCR_012880) [46]. Those proteins having molecular weight of <110KDa were finally designated as potential vaccine candidates as they can be easily purified and can be effectively used for vaccine development [47].

### Epitope mapping of the targeted proteins

The concept of peptide vaccines is based on identification and synthesis of B and T-cell epitopes which are immune-dominant and can generate significant specific immune responses [48]. Immuno-informatics involves various combinatorial computational approaches to predict B and T-cell epitopes [49]. The prioritized proteins obtained by following the above mentioned strategy were subjected to sequential epitope mapping steps. Selection of candidate immunogenic MHC class I and II restricted peptides from characterized proteins is the key to successful vaccine development [50]. ABCpred was employed (threshold value >0.6) to retrieve the B-cell epitopes from the prioritized proteins. ABCpred predicted the B-cell epitopes based on artificial neural networks [51]. The 20-merB-cell epitopes were subsequently analyzed for T-cell epitopes for binding with MHC I and MHC II class molecules by using Proped1 and Proped servers, respectively [52]. The capability of an antigen to generate immune response depends upon its recognition and binding with MHC alleles from both classes I and II [53]. Surface exposure of T-cell epitopes was estimated by using NetSurfP [54]. Vaxijen v2.0 was used to check the antigenicity of the epitopes (threshold value >0.4) [14]. The epitopes having values more than 0.4 were considered potentially antigenic. MHCpred was used to calculate the half maximal inhibition concentration (IC50) score for DRB1*0101 (a common and prevalent allele in worldwide population) [55, 56]. Various *in silico* reverse vaccinology studies targeting bacterial infections, including *A. baumannii*, preferred the epitopes which bind with DRB1*0101 allele, as this will lead to strong antigen recognition and immune response [41, 57, 58]. IC50 values are binding affinity measures calculated from a competitive binding assay [59]. Prediction of MHC binding is a prerequisite to the prediction of T-cell epitopes. Estimation of binding peptides is simplified by following a classification scheme, dividing the peptides into non-binders, low affinity binders (>500nM), medium affinity binders (50–500nM) and high affinity binders (<50nM) [48]. Virulence of the candidate epitopes was estimated by using VirulentPred [60].

### Epitope conservation analysis

A conservation analysis is performed to estimate the level of sequence conservation in the epitopic regions across the included strains of *A. baumannii* (prioritized proteins). The conservation analysis of these epitopes was performed by using CLC main workbench [61]. This user-friendly, graphically based software performs the multiple sequence alignment, and consensus sequence was derived for the epitopes. The overall conservation of the sequences was predicted and displayed with the alignment (Fig. 7). The degree of similarity or variability of a specific protein or epitopic region may provide significant information regarding structural, functional, evolutionary and immunological correlates.

### Protein structure and comparative modeling

Visualization of 3D structure of proteins facilitate in understanding the sequence patterns, functional sites, binding sites and interactions of candidate proteins with other targets [62]. Protein Databank (PDB, RRID:SCR_012820) and Swiss model (SWISS-MODEL Repository, RRID:SCR_013032) were employed to explore 3D structures of the query proteins by comparative modeling [63, 64]. Comparative modeling method make use of experimental protein structures (templates) to build models for target proteins [63, 65]. Swiss model was employed for each of the query protein and among the number of templates generated by the server (BLAST), one with highest sequence identity/similarity was selected. For example, the query sequence adeK exhibited 45.85 % sequence similarity with outer membrane protein OprM (PDB id-3d5k.1.A) which was further assessed for epitope visualization by pepitope. Similarly, for query proteins P pilus assembly protein, pili assembly chaperone, PonA, general secretion pathway protein D (HMPREF0010_02518), FhuE receptor, Type VI secretion system OmpA/MotB, Ton B dependent siderophore receptor, general secretion pathway protein D (HMPREF0010_01958), outer membrane protein, peptidoglycan associated lipoprotein and peptidyl-prolyl cis-trans isomerase, PDB structures with highest sequence similarity were selected, i.e., PDB ID: 3rfz.1.B, 1ze3.1.A, 3udi.1.B,4av2.1.A,2w77.1.A, 2n48.1.A, 1fep.1.A, 4e9j.1.A, 3d5k.1.A, 4g4v.1.A and 1q6u.1.A, respectively. Surface exposure of candidate epitopes on the proteins facilitates in generation of strong immunogenic response [66]. Pepitope was employed to reveal the topology of epitopes within the protein structures [67]. It is essential to ensure the arrangement of the epitopes so that the immunogenic part does not get folded within the globular protein [41].

### Functional annotation of the predicted proteins

Functional analysis assists in studying the biological, molecular and biochemical behavior of the proteins. Here the Blast2GO (RRID:SCR_005828) was employed for functional annotation of the prioritized proteins [68]. Cluster of orthologous groups (COG) and biological/molecular pathways were explored for the prioritized proteins as reported in Kyoto encyclopedia of genes and genomes (KEGG) database (http://weizhongli-lab.org/metagenomic-analysis/server) [69].

### Targeted protein-protein interaction (PPI) analysis

Interaction analysis of prioritized proteins was executed by using Search tool for the retrieval of interacting genes (STRING, RRID:SCR_005223) [70]. The association of query proteins with the predicted functional partners is represented by thick blue lines. Colored nodes represent that the interacting partners directly link to the targeted proteins with optimal score of 0.9. Interactions among the proteins constitute backbone of cellular function and the study of such interactions helps in understanding molecular mechanism and biological processes, elucidating molecular basis of diseases and identifying potential therapeutic targets [71].

### Validation of pipeline through positive control

To test the functionalities of the pipeline and validation of the predictions, additional potential vaccine candidates are predicted against another Gram negative bacterium as a positive control such as *Helicobacter pylori.* The same methodology and pipeline is employed to analyze the genome of *H. pylori* strain reference strain 26695 to find the vaccine targets against this important organism, so as to limit its incidence and eventually the associated gastric cancer.

## Results

### Genome organization and statistics

The drug resistant *A. baumannii* has increasingly become a cause for serious concern with regard to both nosocomial and community acquired infections [4]. Due to next generation sequencing technology, there is wide-ranging availability of genome data on international databases. To date, 30 complete genome sequences of *A. baumannii* are available on GenBank/NCBI, which were all analyzed for potential vaccine candidates in our study (Table 1). The total number of proteins in all of the available complete genomes (30 *A. baumannii* genomes) was calculated as 114,872 and an average *A. baumannii* genome/proteome contain 3,829 proteins. An average GC content is observed around 39.03 %. The average gene count was found out to be 3829 genes (3702 proteins), with lowest number of genes reported in a 50-year old isolate *A. baumannii* ATCC 17978 (3,469) and highest number of genes were reported in a newly sequenced extremely drug resistant *A. baumannii* AB030 (4,336). As a consequence of strong antibiotic selective pressure, the bacteria have acquired significant antibiotic resistance and virulence genes for their survival over the period of time [72]. To get a consistency in the genomic and proteomic data all the sequences were assessed by a single gene prediction program Prodigal which predicted more number of genes in analyzed genome sequences when compared with that of NCBI (Table 1). However, the overall numbers are comparable, this increase in prediction by the Prodigal may be due to its improved translation initiation site recognition [15].

### Virulence potential of *A. baumannii*

Relative to other gram negative pathogens, very little is known in relation to the pathogenic potential and virulence repertoire of *A. baumannii* [73]. To get insight into the virulence potential of *A. baumannii*, virulent genes present in core conserved genome were explored. Interestingly, the bacteria harbor a significant number of virulent genes (295) as part of its core genome which facilitates the organism in pathogenesis and probably survival in adverse conditions (Additional file 1). In the conserved virulome of *A. baumannii* genus, the major virulence factors and associated mechanisms are identified which include OmpA, pili assembly proteins, superoxide dismutase, phospholipases, siderophore dependent iron acquisition proteins, multidrug efflux proteins, and penicillin binding proteins. Among them, the significant protein OmpA is involved in the process of epithelial invasion and apoptosis [74]. Similarly, pili assembly proteins play vital role in bacterial adherence and biofilm formation; thus imperative for pathogenesis of *A. baumannii* [75]. Another important factor, superoxide dismutase found to be present in the core genome assist bacteria by detoxifying reactive oxygen species released in the course of host defense reactions [76]. Like OmpA, the phospholipase C are responsible for survival in human serum and epithelial cell invasion [75]. Upregulated iron acquisition systems and antibiotic efflux pumps further enhance the virulence potential of *A. baumannii* [77].

For a pathogen the virulence mechanism is necessary to overcome the host defense systems and to survive in the competitive and demanding environments (new niches) [78]. Clonal distribution of virulence factors revealed that many of the significant virulent factors are restricted to specific clonal lineages (I and II). For instance, isolates belonging to international clone I (*A. baumannii* AYE, *A. baumannii* A1, *A. baumannii* 0057) exhibits common virulence factors such as Tn21 integrase, streptomycin adenyltransferase, chloramphenicol acetyltransferase, TnmP and transposase. The role of transposon Tn21 in global dissemination of virulence and antibiotic resistant genes have already been explored in Gram negative facultative bacteria [79]. The presence of these virulence determinants in specific clone suggests these are crucial for the success of these widespread common clones. Likewise, in IC II strains, certain virulence factors prevail including phosphomannumatose, type4 pilus assembly protein, heat shock protein 60, tet repressor and tetracycline resistance proteins (Table 2). Presence of important virulence factors in core conserved proteome shows that bacteria has strongly evolved as super bug, and horizontal gene transfer could be answer to this significant phenomenon [80].

**Table 2:** Significant virulence factors in *A. baumannii* strains

Virulence factors	International Clone I				International Clone II						Function
	*A. baumannii*				*A. baumannii*						
	AYE	A1	AB0057	AB307-0294	ACICU	MDRTJ	MDR-ZJ06	TCDC-AB0715	1656	BJAB0868	
Intl1	+	+	+	-	+	+	+	+	+	-	Tn21 integrase
aadA1	+	+	+	-	-	+	+	+			Streptomycin adenyltransferase
Cat	+	+	+	-	-	+	-	-			Chloramphenicol acetyltransferase
tnpM	+	+	+	-	+	+	+	+	-	-	TnmP
tnpA	+	+	+	-	-	-	-	-	-	-	Transposase
tnpH	+	+	+		-	-	-	-	-	-	Hypothetical protein
htpB	-	-	-	+	+	-	+	+	+	+	Heat shock protein60
manB	-	-	-	+	+	-	+	+	+	+	Phosphomannumutase
pilF	-	-	-	-	+	+	-	-	+	+	Type4 pilus assembly protein
tetR	-	-	-	-	-	+	+	+	-	+	Tet repressor
tetAB	-	-	-	-	-	+	+	+	-	+	Tetracycline resistance protein

The signs + and – in the table denote presence or absence of genes, respectively. BLASTp search on Virulence factor database (VFdb) revealed sharing of common virulence factors in *A. baumannii* strains belonging to the international clone I and II. Virulence factors like Tn21 integrase and streptomycin adenyltransferase are frequently shared by strains of both international clone I and II. Transposases (*tnpA, tnpH*) and chloramphenicol acetyltransferases are mostly expressed in strains *A.baumannii* AYE, *A.baumannii* A1 and *A.baumannii* 0057 of IC I. Occurrence of specific virulence factors in IC I and IC II illustrates the evolution of the bacteria as the super bug in certain geographical locations and its survival in localized environments

### Phylogenetic analysis of *A. baumannii strains*

**Fig. 2:**
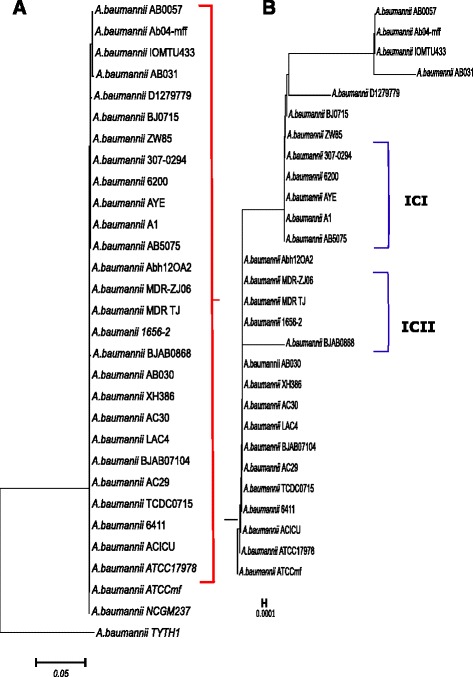
Evolutionary relationships of the *A. baumannii* strains based on 16SrRNA sequences. The evolutionary history was inferred using the Neighbor joining method. 1000 bootstrap re-samplings were done to estimate the consensus tree. The optimal tree with a sum of branch length = 0.5833 is shown. The tree is drawn to scale with branch lengths in same units as those of evolutionary distances used to infer the phylogenetic tree. The evolutionary distances were computed using the maximum composite likelihood method and are in the units of the number of base substitutions per site. **a** Illustrates the complete phylogenetic tree of all the included strains and **b** shows the enhanced view of the selected clads. The strains like *A. baumannii* AYE, *A. baumannii* A1, *A. baumannii* 307-0294 all belonging to IC I fall in the same clade, indicating phylogenetically closeness to their clonal strains. *A. baumannii* D1279779 expressed as a separate branch of the tree, validating the unique properties of this strain in between IC I and IC II. Similarly strains belonging to IC II appeared in single clad, showing their close phylogenetic relationships

Phylogenetic analysis was performed for all the included *A. baumannii* strains to understand the pattern of evolution. The 16SrRNA distance tree illustrates the genetic distances between the closely related sequences. The phylogenetic tree resulted in two main clusters corresponding to IC I (*A. baumannii* AB307-0294, *A. baumannii* AB0057, *A. baumannii* AYE, *A. baumannii* A1) and IC II (*A. baumannii* BJAB07104, *A. baumannii* MDRTJ, *A. baumannii* MDRZJ06, *A. baumannii* TYTH1, *A. baumannii* AC30, *A. baumannii* BJAB0715, *A. baumanni* TCDC, *A. baumannii* AB031, *A. baumannii* ZW85, *A. baumannii* BJAB0868, *A. baumannii* AC29, *A. baumannii* ACICU, *A. baumannii* 1656-2). Selective environmental pressures have led to evolution of strains in specific geographical and ecological niches [81]. *A. baumannii* D1279779 expressed as a separate branch of the tree, validating the unique properties of this strain in between IC I and IC II (Fig. 2) as reported by Ferrugia et al. [82]. The recently isolated bloodstream related *A. baumannii* AB031 exhibited over expression of AdeABC efflux pump, resulting in tigecycline resistance, emerged as a separate branch in the tree [83]. *A. baumannii* IOMTU433 (carrying multiple antibiotic resistance genes notably blaNDM-1, blaOXA-23, blaOXA-104, blaPER-7, armA) [84] and *A. baumannii* Ab04-mff make separate clad indicating they are phylogeneticaly distant from their clonal strains.

### Evolution of genome composition and pangenome

**Fig. 3:**
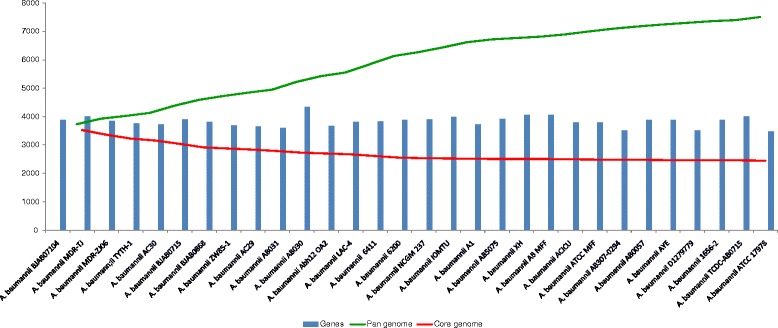
Pan- and core genome plot of *A. baumannii* strains. The X-axis in the graphs denotes all the *A. baumannii* strains included in our study and Y-axis indicates the number of genes expressed in each strain. The green line in the chart indicates the gradual pan-genome expansion and the red line shows the core genome development/conservation with addition of each genome. The blue bars represent the number of genes present in each of the strains. Comparative genomics analysis of all the included strains led us to pan-genome estimation, comprising of 7606 coding sequences, of which 2445 represented core genome. In the pan-core plot, there is a subsequent increase in pan-genome with the addition of next genome, and the core genome decreases with the addition of second genome and so on, till a conserved set of 2445 genes attained representing the core gene repertoire. The stable core genome suggests that it can be explored for identification of conserved vaccine candidates. The gradual extension of the pan-genome with addition of new genome suggests an open pan-genome of *A. baumannii*. The number of genes that each strain contains can be documented from comprehensive statistical analysis given earlier in Table 1

**Fig. 4:**
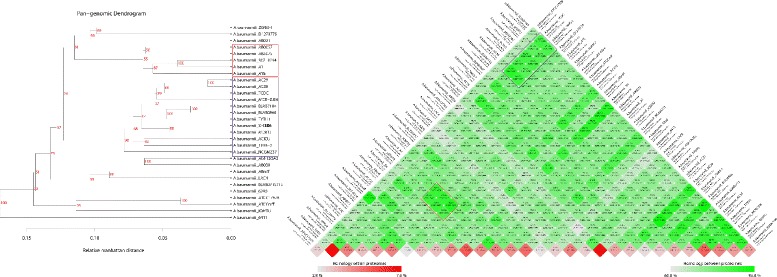
Whole proteome pair-wise comparisons of *A. baumannii* strains. The names of strains along with the number of proteins and protein families are mentioned along each side of the matrix. Each box in the matrix depicts the fractions of shared proteins between the two corresponding strains. The homology between the proteomes is depicted in green color (dark green color represents more homology between proteomes). The number and percentage of shared proteome is also shown in the corresponding box of the matrix. The last row in the matrix depicts the homology within the proteomes and indicated as internal homologies in individual strain (genome/proteome) and are shaded in red color, the scale given showed 2.9 % least to 7.0 % the highest). Two homology blocks (marked in figure as red and blue color) shaded dark green suggest strong homology among the strains. Strains in red and blue block belong to international clone I and II, respectively. The blue block showed homology of more than 80 % between strains *A. baumannii* TCDC and *A. baumannii* 1656-2 with *A. baumannii BJAB7104,* MDR-TJ, MDR-Z, TYTH and AC30. Similarly, in red block three strains (*A. baumannii* AYE, AB0057, AB307-0294) showed more than 83 % homology with *A. baumannii* A1 and *A. baumannii* AB5075. Highest internal homology within proteome was seen in *A. baumannii* strain TCDC and *A. baumannii* AB030 i.e., 7 % and 6.7 % respectively. The matrix results are comparable to pan-core dendogram. Pancore dendrogram is based upon the shared gene families among the strains. IC I and II strains in the red and blue blocks in the matrix, respectively, make separate clads in the tree, indicating the phylogenetic correlation of the strains

Comprehensive pan-genome analysis studies can help to understand the functional adaptation of a bacterial species. In the present study, analysis of *A. baumannii* pan-genome revealed 7,606 coding sequences, of which 2,445 represent core and the remaining 5,161 constitute the dispensable genome. The dispensable genome makes the pan-genome relatively large because of the presence of expansive pool of dispensable genes from various strains. It is observed in the pan-core plot (Fig. 3), that there is a subsequent increase in pan-genome with the addition of new genome, and the core genome decreases with the addition of second genome and so on. We have observed the stability in the core genome while adding third, forth and so on, and finally a conserved set of 2,445 genes was obtained representing the core gene repertoire (Fig. 3) of the selected *A. baumannii* genomes. This core genome is about 64 % of the average genome, which is indicative of the highly conserved genomic features (Additional file 2). Under the selective environmental and antibiotic pressures bacteria acquire genes via horizontal gene transfer, supporting their survival in a specific conditions [85]. The enormous exposure of broad spectrum antibiotics over the years has led this bacterium to acquire foreign genes and up regulate its innate resistance mechanisms [86]. We observed from our analysis that accumulation of an average of 258 new gene families arises in strains, with the highest number of strain specific (unique) new gene families observed were 359, 234, 334, 403 in *A. baumannii* BJAB0715*, A. baumannii* ZW85-1, *A. baumannii* AB030 and *A. baumannii* 6411, respectively (Table 1). Pan-core tree is also generated (Fig. 4) based upon shared gene families between the strains which is a representative tree for the distribution of genomes. The distribution of genomes is found comparable with that of the phylogenetic tree based on 16SrRNA. The findings (pattern of distribution into different clads) are also comparable to whole proteome/blast matrix results. For example, the strain *A. baumannii* TCDC, *A. baumannii* MDR-Z, *A. baumannii* BJAB07104, *A. baumannii* BJAB0868 and *A. baumannii* TYTH-1 are closely related to each other on phylogenetic analysis and also on matrix (Fig. 4) exhibiting more than 80 % similarity in their protein content, with each other.

### Proteome comparison analysis (Intra- and Inter proteome conservation)

The proteome of all the *A. baumannii* strains was compared to estimate the amount of proteins they share. The result was expressed in the form of matrix showing the pair-wise comparison of proteomes (Fig. 4). The number and percentage of shared proteome was shown in corresponding box of the matrix (Fig. 4). The maximum protein conservation i.e., 95.8 %, was found among two carbapenem resistant strains, *A. baumannii* AC29 and *A. baumannii* AC30 strains, both isolated from tertiary care hospital in Malaysia. These two strains belonged to international clone II and had identical Apa I pulsotype [87]. Minimum protein conservation was 60.8 % between *A. baumannii* 6411 and *A. baumannii* AB030 strains, both exhibiting diverse antibiotic resistance genes. *A. baumannii* 6411 possessed blaNDM-1 gene which conferred resistance to carbapenems and cephalosporin, and *A. baumannii* AB030 categorized as extremely drug resistant pathogen (showing resistant to all groups of antibiotics except colistin) [88]. There are certain homology blocks in figure shaded dark green suggest more homology. Block1 (marked in the Fig. 4 as blue colored box) showed homology of more than 80 % between strains *A. baumannii* TCDC and *A. baumannii* 1656-2 with *A. baumannii BJAB7104,* MDR-TJ, MDR-Z, TYTH-1 and AC30, all belonging to IC II. Similarly, in block 2, three strains of IC I (*A. baumannii* AYE, AB0057, AB307-0294) showed more than 83 % homology with other IC I strains, *A. baumannii* A1 and *A. baumannii* AB5075 (marked in the Fig. 4 as a red colored box). The results of the BLAST matrix were found interesting as it can be compared to phylogenetic tree. For example, it can be seen that *A. baumannii* strain TYTH-1 exhibits more than 70 % homology with rest of *A. baumannii* strains and it is seen from the distance tree that these strains have evolved from a common ancestor and share common proteomes. Highest internal homology within proteome was seen in *A. baumannii* strain TCDC and *A. baumannii* AB030 i.e., 7 and 6.7 % respectively (Fig. 4). The relatively high proteome conservation in *A. baumannii* strains, for example 95.8 % homology in two carbapenem resistant strains clinical isolates, make these organism suitable to be targeted for broad spectrum therapeutics and there is a need to estimate the size of the pan-genome of the genus (closely related species).

### Core proteome estimation for essentiality and non-host homologs

**Fig. 5:**
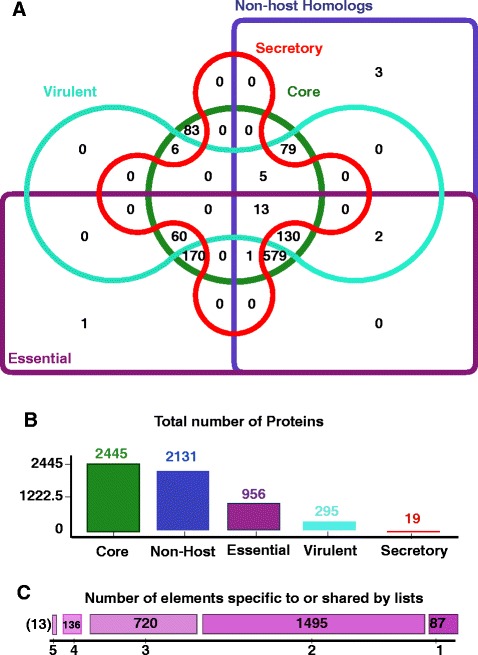
Categorization of core proteome of *A. baumannii.* Illustration of possible correlations between different parameters for the prioritization of core proteome of *A. baumannii* strains. **a** Depicts the generation of the prioritized proteins among the core proteins, selected on the basis of sub-cellular localization, virulence, essentiality and non-host homologs. Core proteins are represented in green circle of the Venn diagram, non-host homologs proteins in blue, essential proteins in purple, virulent proteins in light blue and secreted proteins in red color. The numbers presented in the overlapping regions of the circles show the number of strains exhibiting a particular set of category. 13 proteins among the core proteins have been prioritized, as they simultaneously share all four properties (secreted, essential, virulent, non-host homologs). The proteins satisfying all these criteria were taken into consideration for further epitope mapping approaches. **b** Demonstrates the individual categories i.e., core proteins (green bar, 2,445 proteins), non-host homologs proteins (blue bar, 2,131), essential proteins (purple bar, 956), virulent proteins (light blue bar, 295), and secreted proteins (red bar, 19). **c** Depicts the number of proteins sharing the specific number of attributes (essentiality, virulent, sub-cellular localization, non-host homologs). The scale shifts from dark purple color (1 list) to light color (2, 3, 4 lists)

The identification of essential genes is a crucial step in designing therapeutic targets against bacterial infections because of the fact that most of the antibiotics and vaccines target essential cellular processes [27]. Among the 2445 core proteins, 956 (39 % of core proteome) were predicted as essential genes (blue bar in Fig. 5) (Additional file 3) [89]. Essential genes, comprised of minimal gene set required to support cellular life, have much wider therapeutic potential being antibacterial drug targets and vaccine targets [90]. The essential genes in *A. baumannii* are involved in major biological processes like survival in extreme conditions/stress, infection and persistence in host, nucleic acid binding, ATP and GTP binding, ligase, transferase and phosphatase activity. The core sequences were aligned with human proteome to confirm if there is any similarity between them. Among 2445 core proteins, 2131 proteins (green bar in Fig. 5) showed hits below the threshold value and were considered as non-host bacterial proteins. These non-host proteins/genes are preferred for vaccine development to avoid the generation of autoimmune response or recombination and integration events in the host genome [91]. The details of core proteins, essential, virulent, non-host homologs proteins are provided in Additional file 4.

### Vaccine candidates identification

The presence of core genes in the bacterial genomes is an evidence of conservative nature of evolution [92]. It represents an ideal dataset for the exploration of suitable vaccine candidates against *A. baumannii.* It was subjected to sequential steps in order to find proteins which are at surface or secreted, and at the same time essential, virulent and non host homologs. Proteins prioritized after passing through these criteria were taken as suitable vaccine candidates and were further explored for their epitopes, protein-protein interactions, surface topology, comparative homology modeling and comprehensive functional analysis. The detailed methodology is described in the subsequent sections.

#### Sub-cellular localization

**Fig. 6:**
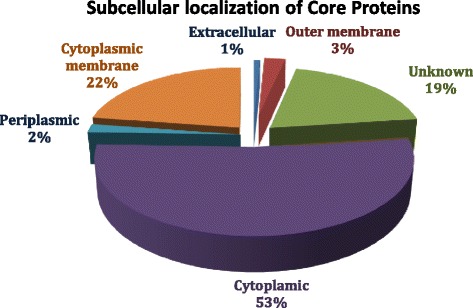
Sub-cellular localization of core proteome for prioritization of vaccine candidates. Core proteins (2445) were analyzed for their sub-cellular localization by using PsortB. A large number (53 %) of proteins were found to be cytoplasmic, followed by 22 % in the cytoplasmic membrane. Proteins situated in outer membrane (3 %), extracellular (1 %) and in periplasmic space (2 %) were preferred to be considered for putative vaccine candidates, as these can be readily recognized by MHC molecules and generate strong immunogenic response

Core proteins estimation for sub-cellular localization revealed that 1289 proteins were cytoplasmic, 552 located on cytoplasmic membrane, 65 outer membrane, 45 periplasmic, 19 extracellular (Additional file 5) [42]. Knowledge of the sub-cellular localization of a protein can significantly improve therapeutic target identification [93]. For example, plasma membrane and secreted proteins are easily accessible by drug molecules due to their localization in the extracellular space or on the cell surface. Bacterial cell surface and secreted proteins are of interest for their potential as vaccine candidates or as diagnostic targets [94] (Fig. 6).

#### Epitope mapping of prioritized proteins

Prioritization and filtration of the identified proteins could help to minimize the time, labor and resources for developing vaccine candidates and optimize the success of getting the best drug or vaccine against the pathogen. Parameters of essentiality, virulent, non host homologs and sub-cellular localization were summed up and 13 core proteins have been observed as potential vaccine targets including P pilus assembly protein, pili assembly chaperone, AdeK, PonA, OmpA, general secretion pathway protein D, FhuE receptor, Type VI secretion system OmpA/MotB, Ton B dependent siderophore receptor, general secretion pathway protein D (HMPREF0010_01958), outer membrane protein, peptidoglycan associated lipoprotein and peptidyl-prolyl cis-trans isomerase. These proteins found out to fit in the criteria of trans-membrane helices (less than 2) and exhibited molecular weight of <110KDa [46]. Antigenicity analysis by using Vaxijen v2.0 revealed that the 13 prioritized proteins are potentially antigenic and exhibited score of more than 0.4 (Table 3). These target proteins were considered as potential vaccine targets and subjected to epitope mapping approaches. The 20-mer B-cell epitopes were predicted for each protein and were further filtered based upon the common T-cell epitopes binding with both MHC I and II molecules (Proped and Proped1). Subsequently, the T-cell epitopes binding to a maximum number of MHC I and II class molecules, with optimal IC50 values for DRB1*0101 were selected. In our study, the ten prioritized proteins were found out as high affinity binders with IC50 values less than 50nM i.e., general secretory protein D (HMPREF0010_01958) (61.94nM), OmpA (1.99nM) ponA (12.76nM), pili assembly chaperone (93.3 nM), general secretion pathway protein D (HMPREF0010_02518) (49.89nM), Ton B dependent siderophore receptor (3.63nM), outer membrane protein (57.68nM), peptidoglycan associated lipoprotein (1.24nM), AdeK (12nM), P pilus assembly protein (11.97nM). Proteins including peptidyl-prolyl cis-trans isomerase (176nM), FhuE receptor (186.64 nM), Type VI secretion system OmpA/MotB (55.72nM) and P pilus assembly protein (267nM) fall in the category of medium affinity binders (Additional file 6).

**Table 3:** Prioritized core vaccine candidates against *Acinetobacter baumannii*

Protein name	T Cell Epitope	Location (aa)	Total number of MHC binding alleles	VaxiJen Score (Antigenicity) (Cut-off value 0.4)	Virulence score (Threshold 0.5)	COG	KEGG Description
P pilus assembly protein (HMPREF0010-00599)	WGDESNERC	820-829	18	1.85	1.0600	COG3188	P pilus assembly protein, porin PapC
adeK	YNSASGTSI	44-52	30	1.9336	1.0621	COG1538	Multidrug resistance outer membrane protein
ompA	YNVDASRLS	298-306	12	1.6125	1.0578	COG2885	ompF-ompA porin family
General secretion pathway protein D (HMPREF0010_01958)	YQQVPSGGK	706-714	7	0.6490	1.0605	COG1450	Bacterial secretion system
FhuE receptor (HMPREF0010-00709)	IKLYDSNVN	641-649	19	1.3939	1.0598	COG4773	Inorganic ion transport and metabolism
General Secretion pathway protein D (HMPREF0010_02518)	IQSSGSYEY	221-229	12	1.1190	1.0606	COG4796	Type II secretory pathway
Type VI secretion system OmpA/MotB (HMPREF0010_01378)	LYLDQKEKK	75-83	9	0.881	1.0607	COG2885	ompF-ompA porin family
Ton B dependent siderophore receptor (HMPREF0010_01517)	YSGDSQLNA	286-294	5	1.4266	1.0636	COG4771	Iron complex Outer membrane receptor
Outer membrane protein (29_170)	IAGNQNLKA	82-90	9	1.8656	1.0596	COG1538	Outer membrane protein
Peptidyl-prolyl cis-trans isomerase (HMPREF0010_03292)	LQTMKEGGK	185-193	4	0.98	1.0610	COG0545	FKBP-type peptidyl-prolyl cis-trans isomerase
Peptidoglycan-associated lipoprotein (HMPREF0010_02142)	YQTLQAHAQ	91-99	17	0.72	1.0606	COG2885	ompF-ompA porin family
Pilus assembly chaperone (HMPREF0010_002598)	IKEDANLAA	159-167	20	0.9917	1.0607	COG3121	Pilus assembly protein
ponA/Penicillin binding protein 1a	FLIIIIILV	15-23	54	2.1254	1.0606	COG5009	Cell wall/membrane biogenesis

Thirteen putative proteins including P pilus assembly protein, pili assembly chaperone, adeK, ponA, ompA, general secretion pathway protein D, FhuE receptor, Type VI secretion system OmpA/MotB, Ton B dependent siderophore receptor, general secretion pathway protein D (HMPREF0010_01958), outer membrane protein, peptidoglycan associated lipoprotein and peptidyl-prolyl cis-trans isomerase have been identified as core vaccine targets by employing *in silico* reverse vaccinology approaches. Antigenic 9-mer T-cell epitopes were identified for each protein by comprehensive epitope mapping strategies. A cluster of orthologous genes (COG) classification and biological/molecular process KEGG description of the potential proteins was determined. Antigenicity and virulence scores were predicted for each of the candidate epitope

The 9-mer sequence YQQVPSGGK from general secretion pathway protein D (HMPREF0010_01958) is surface exposed T-cell eptiope which exhibited antigenicity score of 0.64. The T-cell sequence YNVDASRLS from outer membrane protein OmpA can bind with total of 12 MHC 1 and MHC 11 class molecules, and is highly antigenic (1.61, Vaxijen score) and exhibited the lowest IC50 value for DRB1*0101 allele (1.99nM).The epitope YSGDSQLNA from Ton B dependent siderophore receptor fall in the category of medium affinity binder with seven out of nine surface exposed epitopes and Vaxigen antigenicity score of 1.42. Penicillin binding protein PonA revealed 9-mer completely surface exposed epitope FLIIIIILV with highest anitgenicity score of 2.12 and also bind with maximum number of MHC I and II [54] class molecules. The 9-mer sequence YNSASGTSI from outer membrane protein AdeK can bind with total of 30 MHC class I and II alleles, and is antigenic (1.93, Vaxijen score) and exhibited the lowest IC50 value for DRB1*0101 allele (12nM). Outer membrane protein P pilus assembly protein revealed an epitope WGDESNERC which has Vaxigen score of 1.85, virulence score 1.02, total number of MHC binding alleles 18. Likewise, 9-mer surface exposed T-cell epitopes of pilus assembly chaperone, general secretion pathway protein D (HMPREF0010_02518), Ton B dependent siderophore receptor, outer membrane protein, peptidoglycan associated lipoprotein showed appropriate antigenicity and virulence scores and fulfill all the criteria to be characterized as immunogenic.

### Epitope conservation analysis

**Fig. 7:**
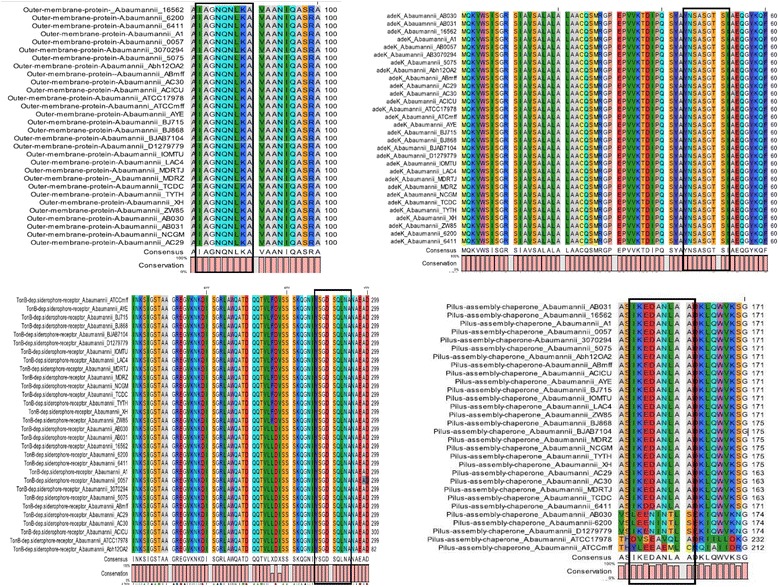
Epitope conservation analysis. The alignments, consensus sequences and the conservation of the epitopes YSGDSQLNA, IAGNQNLKA, IKEDANLAA and YNSASGTSI belonging to proteins TonB dependent siderophore receptor, outer membrane protein, pilus assembly chaperone and adeK, respectively, are highlighted in the Figure

Among the thirteen epitopes in the prioritized proteins, ten epitopes belonging showed 100 % conservation pattern among all of the strains of *A. baumannii* included in the study, these proteins include AdeK, PonA, OmpA, general secretion pathway protein D, Type VI secretion system OmpA/MotB, TonB dependent siderophore receptor, general secretion pathway protein D, outer membrane protein, peptidoglycan associated lipoprotein and peptidyl-prolyl cis-trans isomerase. The alignments, consensus sequences and the conservation in selected epitopes (YNSASGTSI, YSGDSQLNA, IAGNQNLKA, IKEDANLAA) are represented in Fig. 7. Being conserved, these epitopes could be considered as potential core candidates to evoke immune response efficiently within the host against *A. baumannii*. Three epitopes WGDESNERC, IKEDANLAA and IKLYDSNVN have shown conservation of about 80–90 % and these proteins were found to belong to the P pilus assembly protein, pilus assembly chaperone and FhuE receptor, respectively. Hence, the consensus sequence is derived and re-considered for the conservation analysis of the selected epitopic regions; it showed conservation in the same amino acids residues to a great extent. The alignment and conservation of the rest of the epitopes are provided in Additional file 7.

### Prioritized proteins structure analysis

**Fig. 8:**
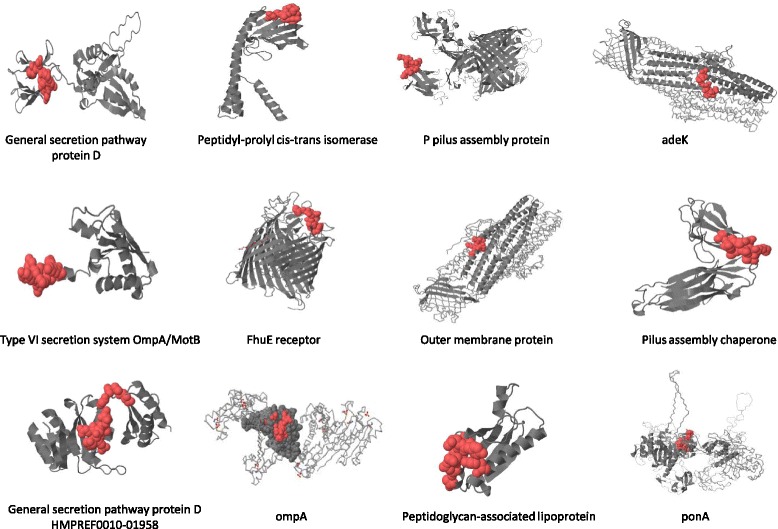
Pepitope depicted surface topology of predicted potential antigenic epitopes. Gray secondary structure of proteins were predicted by using Swiss model and red highlighted structures illustrate the surface topology of 9-mer T-cell epitopes on respective proteins. Surface exposure of epitopes is a requisite for a candidate to be recognized by MHC molecules and be strongly immunogenic. All the predicted vaccine targets fulfill the antigenicity, transmembrane helices and topology criteria and can be exploited further for in vivo testing

The availability of 3D structures of candidate proteins was checked by using protein databank(PDB) [64] and for non-characterized proteins, comparative homology modeling was executed using Swiss-Model [63]. OmpA protein has an experimentally validated 3D structure available on PDB (ID: 4g4y). 9-mer T-cell epitope YNVDASRLS expressed on the surface of OmpA protein (Fig. 7, shown as red spheres on gray colored protein structure), suggesting surface exposure and recognition of the epitope by MHC molecules, resulting in generation of strong immunogenic response. HMMTOP revealed that all the candidate proteins have trans-membrane helices less than 2 and this topological arrangement convincingly support the effective vaccine development [44]. Expression of candidate epitopes on surface of the proteins is imperative, as it would assist in the generation of strong immunogenic response [95]. The selected thirteen epitopes from relevant proteins satisfactorily displayed surface exposure and can be exploited as vaccine targets against *A. baumannii* (Fig. 8) [67].

### Functional annotation of predicted proteins

**Fig. 9:**
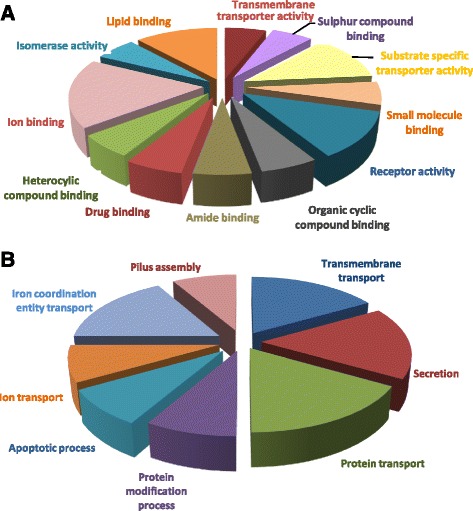
Functional annotation of prioritized protein. Protein function prediction and analysis was executed by using BLAST2GO. **a** Illustrates the involvement of the proteins in significant molecular functions including trans-membrane transporter activity, receptor activity, drug, lipid, ion, sulphur compound and amide binding. **b** Depicts the functional analysis of prioritized proteins based on biological processes. The filtered proteins, being essential and virulent, engage in cell wall biogenesis, secretory system, and assembly of porins, fimbria and receptors, highlighting their importance in cell fitness and survival

Functional analysis of proteins is imperative as it assists in understanding their physiological behavior, biochemical activities and biological processes [96]. The selected proteins were found to be involved in significant biological process including pilus and cell wall organization, chaperone mediated protein folding and transport across membrane. Among them, AdeK is an outer membrane efflux protein, encoded by gene *oprM*, involves in selectively and non-covalently binding with lipids. It enables the directed movement of macromolecules into, out of, within a cell or between cells [77]. Another important factor, OmpA induces apoptosis in laryngeal epithelial cells through capsases dependent and AIF-dependent pathways [97]. It enters the cell and localizes to the mitochondria, and leads to release of pro-apoptotic molecules such as cytochrome C and apotosis inducing factor [98]. Another factor, pili assembly chaperone is engaged in crucial steps of cell wall organization, chaperone mediated protein folding and pilus organization [99]. Similarly, P pilus assembly protein is a fimbrial usher protein and involves in transporter activity [100]. The molecular function of one of the important core vaccine candidate, PonA involves selective and non-covalent binding of any antibacterial drug which contains the condensed beta-lactam thiazolidine ring system [101]. The core vaccine candidate, general secretory pathway protein D is responsible for the secretion of proteins across the outer membrane of bacteria by type II secretion system [102]. At molecular level these protein were found to be involved in transporter activity, antibiotic binding and lipid binding. Another major target, TonB dependent siderophore receptor is located in cell outer membrane. It interacts selectively and non-covalently with iron ions [103]. It is involved in the directed movement of siderophores (low molecular weight iron chelating substances) into, out of or within a cell, or between cells by means of a transporter. Similarly, FhuE receptor is another outer membrane receptor protein required for the uptake of iron via ferric coprogen and ferric-rhodotorulic acid [104]. Another core vaccine candidate, peptidyl-prolyl isomerases (PPIs) are ubiquitous proteins and are involved in the *cis–trans* isomerisation of peptide bonds N-terminal to proline (Pro) residues within polypeptide chains. They are significantly engaged in the folding of newly synthesized proteins and in the function of the immune system [105]. B2GO facilitate in mapping and graphical representation of the results (Fig. 9). COG analysis showed that the candidate seven proteins belonged to significant groups (Additional file 8) [69]. Prioritized targets were found to be involved in the major biological and molecular pathways as reported in KEGG database (Additional file 9) [69, 106]. The filtered proteins, being essential and virulent, engage in cell wall biogenesis, secretory system, and assembly of porins, fimbria and receptors, thus highlighting their importance in cell fitness and survival.

### Protein-protein interactions (PPI) analysis of the candidate proteins

**Fig. 10:**
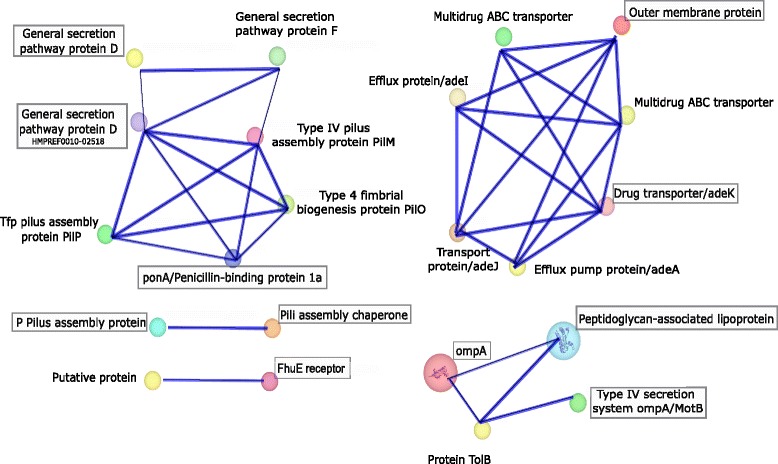
Protein-protein interactions. Stronger associations are represented by blue thick lines. The predicted functional partners for our query protein adeK are a family of adeA, adeJ, adeI, multidrug ABC transporter and multidrug efflux pump. Pili assembly chaperone and P Pilus assembly protein, which are responsible for pili assembly have interaction with a fimbrial protein. Outer membrane protein OmpA interacts with TolB (involved in TonB-independent uptake of colicins) and peptidoglycan associated lipoprotein. ponA (penicillin binding protein 1a) and general secretion pathway protein D interacts with a group of Type IV pili proteins, including Tfp pilus assembly protein PilP, type IV fimbrial biogenesis protein PilO and type IV pilus assembly protein PilM. These interactions show that the bacteria possess flagellum independent surface associated motility, and ponA and general secretion pathway protein D assist the type IV pili multi-protein in exhibiting this property in *A. baumannii.* These associations aid bacteria to adhere to biotic and abiotic surfaces and facilitate in enhanced virulence, colonization and subsequently to disease

Proteins in a biological system interact not only with each other but also with other molecules that mediates metabolic/signaling pathways and cellular processes [107]. Predicted thirteen proteins were found to be interrelated with the important proteins like efflux pump proteins, multidrug ABC transporters and multidrug resistant proteins (Fig. 10). Four clusters were established exhibiting involvement of candidate proteins in significant biological processes like transport, cell wall biogenesis and binding (Fig. 9). PonA (penicillin binding protein 1a) and general secretion pathway protein D (HMPREF0010-02518) interacts with a group of Type IV pili proteins including Tfp pilus assembly protein PilP, type IV fimbrial biogenesis protein PilO and type IV pilus assembly protein PilM. These interactions suggest that the bacteria possess flagellum independent surface associated motility, and PonA and general secretion pathway protein D assist the type IV pili multi-proteins in exhibiting this property in *A. baumannii* [108]. P pilus assembly protein and pili assembly chaperone make a separate cluster and interact with other pili assembly chaperones and fimbrial proteins. In a separate cluster, AdeK (outer membrane efflux protein oprM) has close interactions with large family of AdeI, AdeJ, AdeA and multidrug ABC transporter [109]. Another of our core priortized protein FhuE receptor closely interacts with Ton-B dependent siderophore receptor, indicating the dual mechanism which facilitates bacteria to survive in iron-limiting conditions [110]. In another cluster, OmpA, peptidoglycan associated protein and Type VI secretion system OmpA/MotB interacts with *tolB* which is involved in TonB independent uptake of molecules [111]. By analyzing the PPI network graphs, genetic/metabolic correlations and the co-localizations of the proteins were determined. Protein interaction networks facilitated in understanding the evolution of individual proteins and the different systems in which they were involved. These helped to suggest functions for the proteins by revealing their roles in pathways and protein complexes [112].

#### Validation of pipeline through positive control

*H. pylori* genome subjected to the same prediction process revealed five significant vaccine candidates which includes vacA, babA, sabA, fecA and omp16. The pipeline successfully identified already known immunogenic targets (vacA) [113] along with novel ones (babA, sabA, fecA and omp16) [114] against this Gram negative pathogen.

## Discussion

*A. baumannii* is an emerging MDR pathogen which is responsible for 2–10 % of all Gram negative hospital infections [115]. The devastating healthcare and economic impact of *A. baumannii* infections in hospitals worldwide emphasize on the imperative need to exploit new approaches to confront the said pathogen. We have employed the pan-genomics and reverse vaccinology approaches which have revolutionized the understanding and tackling bacteria over the recent years. In this study, 30 complete genomes of *A. baumannii* were analyzed within the framework of pan-genomics, comparative genomics and proteomics. Previously, *A. baumannii* comparative studies employed varying sample sizes to study the pathogen genomics. Sahl et al. executed pan-genome analysis of six complete genomes [116]; Di Nocera et al. compared seven strains responsible for nosocomial outbreaks in Mediterranean hospitals [117]. A wider and comprehensive analysis based on all completely sequenced genomes would provide a better framework for comparison than few organisms. This study showed the subsequent increase in pan-genome size with the addition of new genomes which is suggestive of an open pan-genome in *A. baumannii* and emphasizes on the presence of gene acquisition and loss events in the evolution, adaptation and persistence of this human pathogen [118]. The pan-genome comprised of 7,606 coding sequences, of which 2,445 represent core and 5,161 were dispensable genome. The pan-genome appeared to be remarkably large because of presence of expansive pool of dispensable genes. The diverse pan-genome is suggestive of frequent horizontal gene transfer events [119]. The evolution of pan-genome is largely affected by the process of gene conservation and transfer. Various studies have debated on the large and open pan-genome of *A. baumannii* [120, 121]. Few comparative genomic studies targeted the pan-genome of *A. baumannii* and subsequently the core genes have explored 1,455 and 2,688 coding sequences depending on the number and identity of strains analysis [82, 118, 122]. The open pan-genome suggests that the said pathogen has the remarkable propensity for gene gain and gene loss, which could help in its survival in diverse ecological niches, and could evolve with enhanced pathogenicity [118].

The *A. baumannii* strains have been classified based upon their clonal lineage to get better insights into epidemiology of this human pathogen [123]. The rationale for exploring the clonal lineage and sequence typing facilitate in characterization of multidrug resistance phenotype among various strains. Secondly, these clonal studies assist in understanding the phenomenon of horizontal gene transfer in the *A. baumannii* strains, persuading in adaptation of this bacterium in diverse ecological environments [19]. For instance, *A. baumannii* AYE belonging to clonal complex I had acquired an 86-kb resistance island (AbaR) under the selective pressure of broad spectrum antibiotics. AbaR is absent from first sequenced *A. baumannii* ATCC 17978, isolated before the development of new generation antibiotics [118]. The phylogenetic analysis based on 16srRNA sequences and on shared gene families showed that strains belonging to same clonal lineages are closely related to each other and they exhibit strong homology in their proteome. The comprehensive whole proteome pair wise comparison led us to similar findings. The presence of common proteins among the strains convincingly supports that peptide vaccines can be efficiently developed against *A. baumannii* [124].

The success of *A. baumannii* can be attributed to its remarkable ability to harbor significant virulence factors as part of its core conserved genome; aiding it to adhere, colonize, form biofilms and escape antibiotics [125]. Existence of certain virulence factors in the core genome like peroxiredoxin, high temperature protein G (HtpG), thioredoxin disulfide reductase (TrxB), superoxide dismutase, chaperone protein DnaJ, support *A. baumannii* to survive in stressful conditions. Furthermore, it was observed that there is spread of similar virulence factors and among a single clonal complex, thus aiding in the persistence of this bacterium in a particular geographical location. The existence and expansion of homogenous clonal lineages, whose main difference from the non-clonal *A. baumannii* appears to be their antimicrobial resistance, credibly suggests that there is horizontal acquisition of resistance genes from other nosocomial pathogens [126]. Virulence factors comprised of 12 % of the core genome, which suggests that acquisition or existence of virulence factors is not likely a predominant factor in the recent nosocomial spread of *A. baumannii* clones. Other factors should be considered for the evolution of this pathogen as global superbug. One possibility is its innate notorious ability to adapt to its adverse environmental conditions and nosocomial settings. Another likelihood could be the differential regulation of conserved set of virulence genes according to the strain [127]. Synergistic effect of multiple genes or polymorphic differences in shared virulence genes may play a part in diverse virulence potential of said pathogen [86].

Due to its remarkable ability to develop antimicrobial resistance, vaccination is considered a reliable alternative strategy to prevent infections caused by this drug resistant bacterium [128]. Few conventional approaches have previously been used to identify potential vaccine candidates. Outer membrane and whole cell preparations have been employed in murine models of sepsis and generated active and passive immune response [129, 130]. But endotoxin contamination limited these conventional approaches to be developed or used in humans. In contrast, development of subunit peptide vaccines based on *in silico* reverse vaccinology (RV) approaches offer a feasible alternative, as these can induce efficient immunogenic response and can be obtained on a large scale [9]. The use of sequence based approaches ensures the reliable vaccine candidates, and is strengthened by the employment of various search tools and filtering steps [131]. In this study, we have adopted combinatory approach to identify putative vaccine candidates against *A. baumannii*, and we deduce that this will be more efficient than data obtained from each method as a standalone technique. Core protein of all the 30 included strains was considered as it would lead to a more representative and conserved set of targets. The strains employed covered representatives from the international *A. baumannii* clones currently circulating the globe. Appropriate location of proteins aid in effective recognition by MHC molecules and in inducing strong immunogenic responses [132]. Among the core proteins, we have selected those proteins which are on cell surface or in periplasmic or extracellular space. Parameters of essentiality, virulent, non-host homologs, molecular weight and trans-membrane helices were summed up and thirteen core proteins have been found out to be potential vaccine candidates. This included OmpA which have been previously examined as vaccine candidate against *A. baumannii.* OmpA is an important virulence factor in the pathogenesis of *A. baumannii* infections [133]. OmpA is highly conserved among the clinical strains, but share minimal homology to human proteome [134]. In vitro studies have shown that it induces phenotypic maturation of dendritic cells (DC) and promote Th1 immune responses, and in vivo murine melanoma models, it stimulates maturation of murine splenic DCs and associate with enhanced surface expression of co-stimulatory molecules CD 80 and CD 86 and MHC class I and II molecules of dendritic cells [135]. In another in vivo testing of OmpA antigen with aluminium hydroxide adjuvant in diabetic mice resulted in production of high anti-OmpA antibody titers and also improved survival of mice following intravenous infection with *A. baumannii* [134, 136]. In another study, OmpA was found to induce little protection in a mouse model of *A. baumannii* pneumonia infection [137]. The difference in these observations could be due to route of challenge, immunization strategies, OmpA refolding, and animal models. Besides, OmpA few other antigens have been identified which could elicit immune response. By combining *in silico* comparative genome analysis with proteomic approaches, Moriel et al. identified 42 surface-exposed and secreted antigens from *A. baumannii* that could be used as potential vaccine targets [138]. Chiang et al. analyzed 14 complete genomes of *A.baumannii* and identified 13 genes from 2752 homologous core genes of *A. baumannii* as potential vaccine candidate antigens. Peptidoglycan associated lipoprotein identified from our analysis of 30 complete genomes was also identified as potential antigenic candidate by Chiang et al. [139].

It is analyzed in this study that two of the prioritized core proteins PonA and general secretion pathway protein D interact with three TFP proteins naming Tfp pilus assembly protein PilP, type 4 fimbrial biogenesis protein PilO and type IV pilus assembly protein PilM. These three TFP proteins are bacterial surface appendages and participate in natural transformation, twitching motility and adherence of bacteria [140]. This suggests that bacteria possess significant surface core proteins which assist with TFP proteins to help bacteria to exhibit motility. This property helps bacteria to adhere to biotic and abiotic surfaces and facilitate in virulence, colonization and subsequently cause the infection. Peleg et al. recently studied the virulence behavior of six *A. baumannii* strains and interestingly found presence of TFP proteins in its core proteome, facilitating in survival of bacteria in divers ecological niches [141]. Recently, Eijekelkamp et al. observed that all the *A. baumannii* strains that belong to clonal lineage I were capable of twitching motility, indicating that they may produce TFP [142]. AdeK, another core vaccine target in this study, belongs to AdeIJK resistance nodulation cell division (RND) family efflux pump reported in *A. baumannii* [143]. This tripartite pump comprised of AdeI, AdeJ and AdeK genes which encode membrane fusion protein, transporter and outer membrane component of the pump, respectively [143]. This type of pumps contributes to resistance to β -lactams, chloramphenicol, tetracycline, erythromycin, flouroquinolones, fusidic acid, trimethoprim, rifampin, pyronine and sodium dodecyl sulphate [144]. Presence of AdeK in core proteome of thirty *A. baumannii* strains suggests that bacteria have acquired robust mechanisms in its core genome to exhibit resistance to almost all major antibiotics. PPI showed that this core protein interacts with its accomplices (AdeI, AdeJ, AdeK) and with multidrug transporter proteins enabling it to successfully efflux antibiotics, leading to its survival in hospital settings. TonB dependent siderophore receptor found out as an essential core protein in our study. Cells growing in aerobic conditions have adapted complex strategies to overcome scarcity of iron, an essential element. Outer membrane protein localized complexes like TonB proteins bind with iron chelates at the cell surface and promote their uptake [145]. We have observed that *A. baumannii* correlates with FhuE receptor for maximal transport efficiency of iron, coprogen and ferric-rhodotorulic acid across the membrane [104]. Fajardo Bonin et al. used human sera from *A. baumannii* infected patients to screen against *A. baumannii* outer membrane proteins and identified six immune-reactive proteins including ferric siderophore receptor protein, OmpA, Omp34kDa, OprC, OprB-like, OXA-23 [146, 147]. Further in vivo studies are required to validate the immunogenicity of these candidates.

The conserved regions within a protein are considered to evolve slowly. These regions usually considered vital for function and believed to be associated with lower variability and are highly conserved among different strains, additionally, these regions are often represent reliable targets for the development of epitope-based vaccines [148]. Likewise, the predicted vaccine candidates containing conserved (100 %) epitopes such as IQSSGSYEY, YSGDSQLNA might be effective in providing broad-spectrum protection. The three epitopes (WGDESNERC, IKEDANLAA and IKLYDSNVN) showed 80–90 % conservations which is an indication of a slight variability in parent proteins and hence the rapid evolution the regions. As these epitopes are found with less than the identity level threshold and should be critically re-considered as they may suggest the uniqueness of the specific epitope. As the different strains of *A. baumannii* included in this study belong to different geographical locations they might have adapted diverse virulence mechanism and pathogenesis, such as proteins P pilus assembly protein, pilus assembly chaperone and FhuE receptor. These proteins and associated epitopes may be effectively considered for designing strain-specific vaccines.

The pipeline is validated by predicting the potential vaccine candidates against *H. pylori*. Studies on large and small animal models have already been carried out to demonstrate the efficacy of vacA and cagA against *H. pylori* through vaccination [149, 150]. In addition, promising findings were also observed in preclinical trials as a result of vaccination, which includes the induction of T-cell mediated immunity (local gastric Th1 and Th17 responses) [151]. Two recently conducted phase I clinical trials in human volunteers based on three recombinant antigens (cagA, vacA and NAP) induced T-cell responses [152]. Our computational pipeline successfully identified already known immunogenic targets (vacA) [149, 153] and along with novel ones (babA, sabA, fecA and omp16) [114] against the *H. pylori.* This serves as positive control for our proposed pipeline to identify potential vaccine targets in *H. pylori*. Thus, we are confident about the predictions by employed methodology and the identified vaccine candidates against *A. baumannii* and hence the functionalities of the proposed pipeline.

All the theoretical approaches have advantages and limitations in general, regarding the methodology adopted in this manuscript; a possible challenge is the predicted prioritized protein “PonA (PBP1a)” which is found to be attached with the cytoplasmic membrane and hence, may not be effectively generate immune response (antibodies). Basically, parameters were set to include all the outer membrane proteins, extracellular and periplasmic proteins. Among the predicted proteins, ponA is found out as extracellular protein by the sub-cellular localization program “PsortB”. This could be considered as a limitation of the tool itself, not with the overall pipeline; therefore it is suggested that the researcher should cross-check the findings with other subcellular localization tools such as Cello, MetaLocGramN etc. [93, 154]. In this manuscript, a universal pipeline is proposed which could be applied on both the Gram positive and Gram negative bacteria in general; however, it is strongly suggested that the parameters may be carefully monitored and adjusted while dealing with Gram negative bacteria due to compositions of cell wall.

Similarly, the peptidoglycan associated lipoprotein which is anchored in the outer membrane of Gram negative bacteria through an N-terminal hydrophobic domain and interacts with the cell wall peptidoglycan by C-terminal OmpA-like domain. This protein is classified as an outer membrane by the program (PsortB) employed for subcellular localization and validated by another program (Cello) as well. Previously, the peptidoglycan associated lipoproteins have been reported to be potential vaccine candidates in few Gram negative bacteria e.g., *Haemophilus influenzae, Haemophilus ducreyi, Legionella pneumophila and Campylobacter jejuni*. Quite recently, in a study conducted by Huang et al. in 2016, Omp22 from *A. baumannii* efficiently elicited high titers of specific IgG in mice [155]. Domain analysis revealed that Omp22 might be an “outer membrane protein-related peptidoglycan-associated (lipo) protein” [155]. This in vivo study further strengthens the role of this protein in generating substantial immune response against *A. baumannii.*

Considerable progresses have been made recently in the development of an effective vaccine to combat infections due to MDR *A. baumannii*. Several experimental vaccines have been evaluated and induced satisfactory antibody responses [156]. However, substantial methodical challenges remain in the development of safe and effective vaccines for *A. baumannii.* The role of antibodies in vaccine induced protection has already been elucidated in immunization studies but thorough advanced specifics like antibody mediated opsonization, bactericidal studies, mucosal IgA antibody response, T cell response need further research. These understandings will likely to accelerate the vaccine development. Based upon the rapid course of *A. baumannii* infection, it is imperative to have an efficient vaccine which can control bacterial replication at an early stage of disease. Compared to other pathogens, relatively smaller numbers of antigen candidates have currently been identified for *A. baumannii* vaccines. However, recent enhanced exploration of pan-genomics, virulence factors, antibiotic resistant islands, pathogenicity islands of *A. baumannii* are likely to expedite the identification and development of effective vaccine candidate against said pathogen. Large scale studies like ours encompassing the pan-genomics, comparative genomics, reverse vaccinology, immunoproteomics would contribute to rational design of vaccine against *A. baumannii.*

## Conclusion

The study, based upon combinatorial approach of pan-genomics, core genomics, proteomics and reverse vaccinology led us to find out potential vaccine candidates against *A. baumannii.* The comprehensive analysis of all the completely sequenced genomes revealed thirteen putative antigens naming P pilus assembly protein, pili assembly chaperone, AdeK, PonA, OmpA, general secretion pathway protein D, FhuE receptor, Type VI secretion system OmpA/MotB, TonB dependent siderophore receptor, general secretion pathway protein D, outer membrane protein, peptidoglycan associated lipoprotein and peptidyl-prolyl cis-trans isomerase;which could elicit substantial immune response. The integration of computational vaccinology strategies would facilitate in tackling the rapid dissemination of resistant *A.baumannii* strains. Future work is suggested towards the characterization of the candidate proteins and immunology followed by evaluation in animal models.

## Abbreviations

*A. baumannii*, *Acinetobacter baumannii*; BLAST, Basic Local Alignment Search Tool; BLASTp, Protein BLAST; COG, Cluster of Orthologous groups; DEG, Database for Essential genes; IC50, Half maximal inhibition concentration; IDSA, Infectious Diseases Society of America; KEGG, Kyoto encyclopedia of genes and genomes; MHC, Major histocompatibility complex; MLST, Multi locus sequence typing; MvirDB, Microbial virulence data base; NCBI, National Center for Biotechnology Information; PDB, Protein databank; VFDB, Virulence factor database

## Additional files

Additional file 1: Virulent protein in core proteome: Virulent proteins (295) present in core conserved genome (2445) of *A. baumannii* are identified, and found out to facilitate the bacteria in its pathogenesis. (XLSX 123 kb) Additional file 2: Core proteins: Fasta sequences of 2445 core proteins are mentioned. (XLSX 550 kb) Additional file 3: Essential proteins: List of essential proteins (956) identified by employing DEG. (XLSX 98 kb) Additional file 4: Lists of core, essential, virulent and non-host homologs proteins: Parameters of essentiality, virulent, non host homologs and sub-cellular localization were summed up from the core proteins and 13 core proteins have been found out be potential vaccine targets. (XLSX 69 kb) Additional file 5: PsortB sub-cellular localization of core proteins. (XLSX 480 kb) Additional file 6: Eptiope analysis of 13 prioritized proteins. B-cell epitope predictions, T-cell epitope location, antigenicity, NetsurfP exposure of epitopes. (XLSX 63 kb) Additional file 7: Epitope conservation analysis of WGDESNERC, YNVDASRLS, YQQVPSGGK, IKLYDSNVN, IQSSGSYEY, LYLDQKEKK, LQTMKEGGK, YQTLQAHAQ and FLIIIIILV belonging to proteins P pilus assembly protein, ompA, General secretion pathway protein D, FhuE receptor, general secretion pathway protein D(HMPREF0010_02518), type VI secretion system OmpA/MotB, peptidyl-prolyl cis-trans isomerase, peptidoglycan-associated lipoprotein and ponA, respectively. (PDF 1475 kb) Additional file 8: COG analysis of core proteins. (XLSX 224 kb) Additional file 9: KEGG description of core proteins. (XLSX 515 kb)
