# Supplementary figures and images for: Pangenome and immuno-proteomics analysis of Acinetobacter baumannii strains revealed the core peptide vaccine targets
Source: BMC Genomics. 2016 Sep 15;17:732. doi: 10.1186/s12864-016-2951-4 (PMC5025611; doi:10.1186/s12864-016-2951-4)

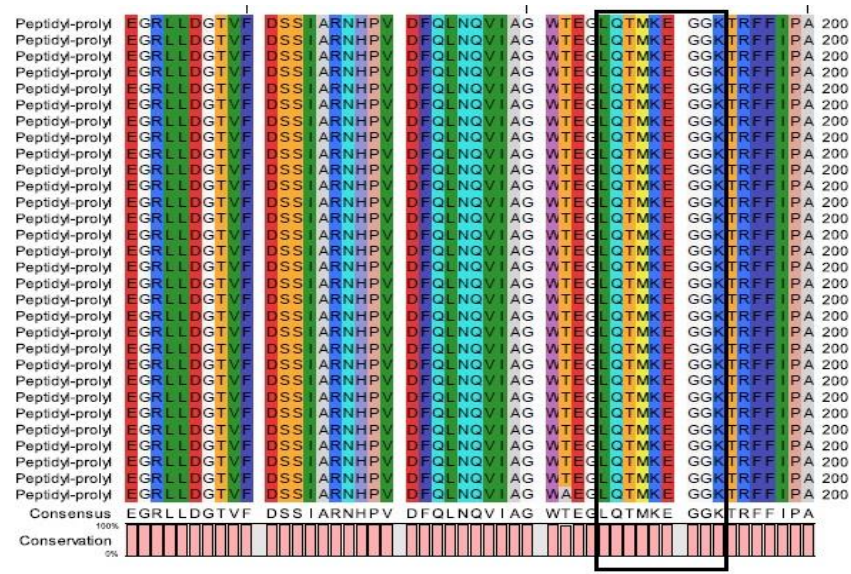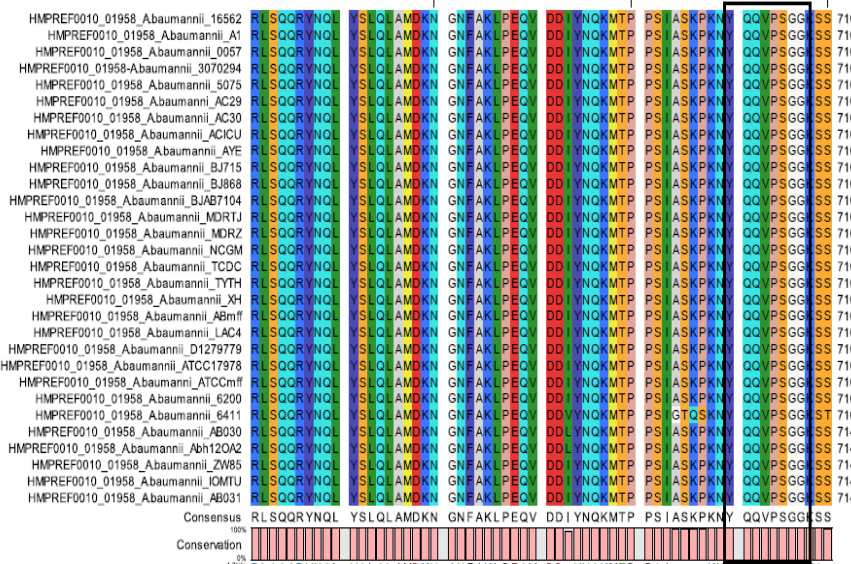

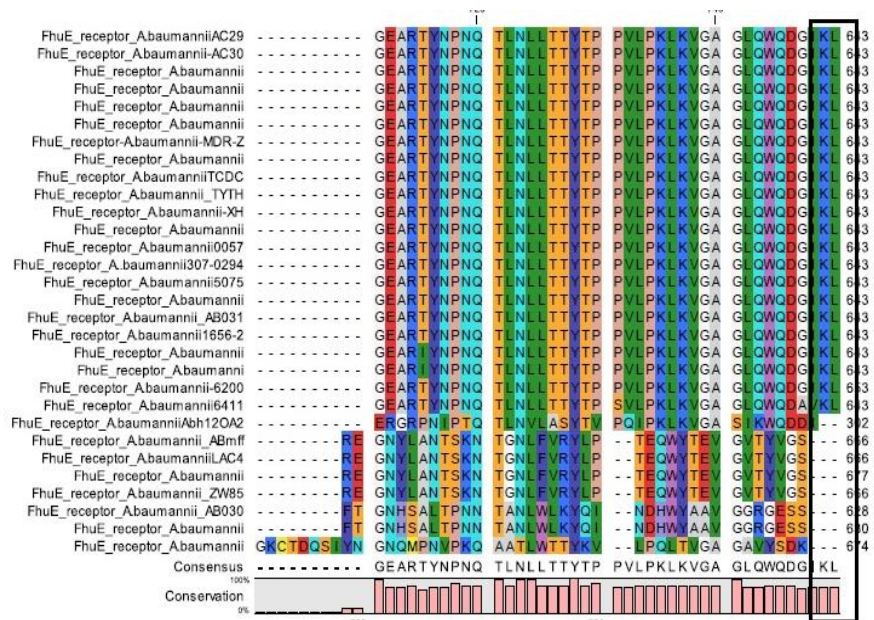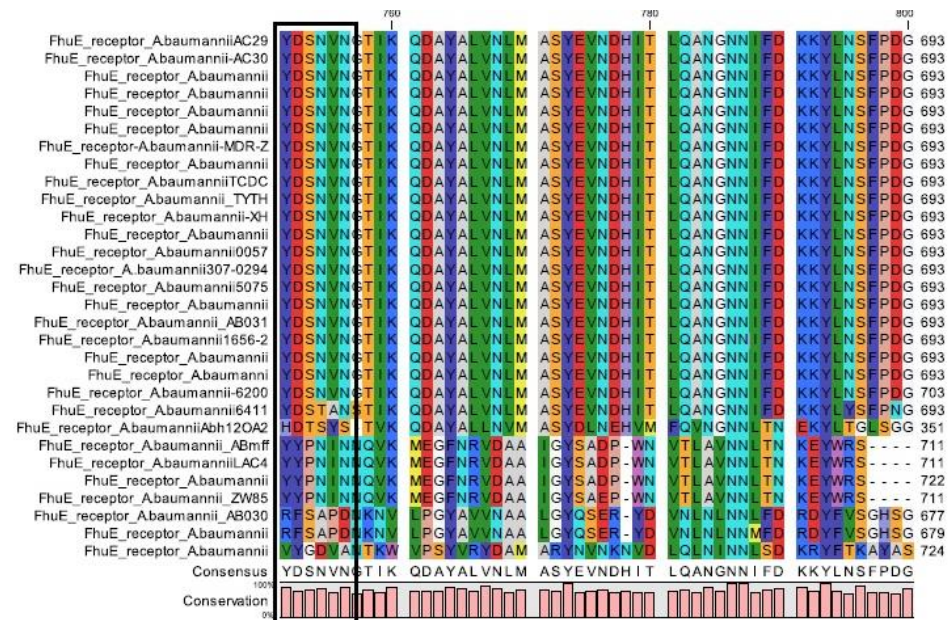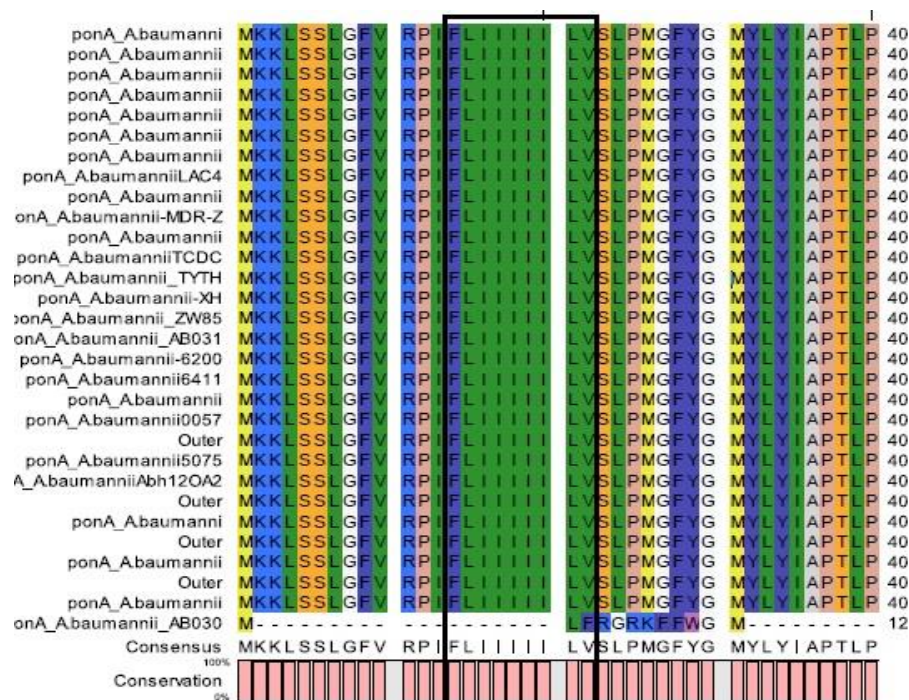

Supplement: Additional file 7: — Epitope conservation analysis of WGDESNERC, YNVDASRLS, YQQVPSGGK, IKLYDSNVN, IQSSGSYEY, LYLDQKEKK, LQTMKEGGK, YQTLQAHAQ and FLIIIIILV belonging to proteins P pilus assembly protein, ompA, General secretion pathway protein D, FhuE receptor, general secretion pathway protein D(HMPREF0010_02518), type VI secretion system OmpA/MotB, peptidyl-prolyl cis-trans isomerase, peptidoglycan-associated lipoprotein and ponA, respectively. (PDF 1475 kb) [file 12864_2016_2951_MOESM7_ESM.pdf]
